# Hearing Loss and Risk Factors in Very Low Birth Weight Infants

**DOI:** 10.3390/jcm12247583

**Published:** 2023-12-08

**Authors:** Simonetta Frezza, Eloisa Tiberi, Mirta Corsello, Francesca Priolo, Francesco Cota, Piero Catenazzi, Guido Conti, Simonetta Costa, Giovanni Vento

**Affiliations:** 1 Department of Woman and Child Health and Public Health, Catholic University of Sacred Heart, Fondazione Policlinico Universitario A. Gemelli IRCCS, 00168 Rome, Italy; simonetta.frezza@unicatt.it (S.F.); eloisa.tiberi@policlinicogemelli.it (E.T.); francesca.priolo@policlinicogemelli.it (F.P.); francesco.cota@policlinicogemelli.it (F.C.); simonetta.costa@policlinicogemelli.it (S.C.); giovanni.vento@unicatt.it (G.V.); 2 Neonatal Intensive Care Unit, Maggiore Hospital, 40133 Bologna, Italy; piero.catenazzi@ausl.bologna.it; 3 Department of Head and Neck Surgery, Clinic of Otorhinolaryngology—Audiology Service, Catholic University of Sacred Heart, Fondazione Policlinico Universitario A. Gemelli IRCCS, 00168 Rome, Italy; guido.conti@unicatt.it

**Keywords:** very low birth weight infants, sensorineural hearing loss, neonatal screenings, ototoxic drugs, neonatal intensive care unit

## Abstract

The incidence of sensorineural hearing loss (SNHL) is still high in very low birth weight (VLBW) infants. The purpose of our study was to provide the prevalence rates of SNHL and to analyze the risk factors of hearing impairment and changes in hearing thresholds in a cohort of VLBW infants. A retrospective observational study was conducted in our neonatal intensive care unit (NICU) from 2012 to 2016. All VLBW infants included were screened by transient evoked otoacoustic emissions (TEOAEs) and diagnostic auditory brainstem response (ABR). In total, we enrolled 316 infants and SNHL was diagnosed in 68, leading to an early incidence of 21.5% as 36 infants out of 68 improved. Finally, SNHL was confirmed in 20 patients (6.3%) who needed hearing aids. They were significantly smaller, sicker, had longer hospitalizations, and received more ototoxic therapies. Logistic regression analysis showed that gestational age (GA) influenced the association between drugs and SNHL. The results underlined how the total exposure to antibiotics is significantly associated with SNHL, even after GA correction. In conclusion, GA, birth weight and, above all, the length and complexity of NICU stay quantify the risk of SNHL and should be considered at the individual level for parent counseling.

## 1. Introduction

In the last ten years, the survival rate of extremely preterm infants has greatly improved. Despite this advancement, the incidence of neurodevelopmental and sensory problems remained unchanged. Sensorineural hearing loss (SNHL) affects 2–4% of babies admitted to the neonatal intensive care unit (NICU) [1]. This prevalence seems to be higher among very preterm infants, reaching 15% [2,3].

Universal newborn hearing screening (UNHS) routinely allows an early diagnosis and the activation of a targeted treatment of SNHL, aimed to ensure adequate development of speech and language, as well as intellectual and emotional growth [4,5].

The Joint Committee on Infants Hearing (JCIH) program for UNHS reported the risk factors for hearing impairment in childhood [6,7].

Genetic etiology accounts for 50–60% of permanent hearing loss in childhood, while 25–30% of cases are due to non-genetic acquired factors. However, the etiology of hearing loss still remains unknown in a large percentage of cases [8,9]. Prenatal and postnatal infections, ototoxic agents, noise, prolonged mechanical ventilation, asphyxia and, above all, prematurity and low birthweight (BW) are generally associated with admission to NICU [10,11], and it is unclear which of these factors has an independent role in causing hearing loss (HL) [12,13].

Although hearing impairment in newborns has been investigated in several studies, there are few authors who carefully analyzed the correlation between HL and prematurity, and there is no uniformity with regard to the testing methods, the time of audiological diagnosis and the management of infants during the first months of life [13].

The aim of our study is to provide the prevalence rates of SNHL in a cohort of very low birth weight (VLBW) infants and to analyze the risk factors for hearing impairment in order to propose more efficient and specific modalities for audiological intervention and communication with family. For this purpose, the changes in hearing threshold observed during the audiological follow-up were also analyzed.

## 2. Materials and Methods

### 2.1. Study Design

This was a retrospective two-group comparative observational study. The study was approved by the institutional review board of the Fondazione Policlinico Universitario A. Gemelli IRCCS, Rome, Italy (Protocol ID 4927) and was performed in accordance with the Declaration of Helsinki. Written informed consent was obtained from the parents of all patients included in the study.

### 2.2. Setting, Inclusion and Exclusion Criteria

From January 2012 to December 2016, all preterm infants with BW < 1500 g were admitted to our NICU, screened by means of transient evoked otoacoustic emissions (TEOAEs) before discharge and after 32 weeks of postmenstrual age (PMA) and by means of diagnostic (laboratory recording) auditory brainstem response (ABR) within 3 months of corrected age (CA). Infants with congenital infections, dysmorphic features, craniofacial abnormalities and without informed consent were excluded.

### 2.3. Primary and Secondary Outcomes

The primary outcome was the incidence of SNHL in our cohort of VLBW infants. Secondary outcomes were to identify the potential independent risk factors of SNHL and to analyze the changes in the hearing threshold during the course of the audiological follow-up.

### 2.4. Data Collection and Definitions

We considered the following variables: BW; GA determined by the best obstetric estimate on the basis of the first day of the last menstrual period, prenatal ultrasound and postnatal physical examination; gender; small for GA (SGA) infants, defined with the Italian Neonatal Study (INeS) reference values as those whose BW z-score was below −1.28 standard deviation (SD) [14]; and Apgar score.

Among morbidities, we recorded the presence of hemodynamically significant patent ductus arteriosus (hsPDA) requiring medical or surgical treatment, the presence of bronchopulmonary dysplasia (BPD) [15], brain injuries such as intraventricular hemorrhage (IVH) > grade II [16] or cystic periventricular leukomalacia (PVL) ≥ grade II [17], retinopathy of prematurity (ROP) [18] requiring surgical treatment, major gastrointestinal surgery, hyperbilirubinemia needing exchange transfusion and proven sepsis, defined as a positive blood and/or cerebrospinal liquor culture together with clinical features.

Among treatments, oxygen therapy, invasive mechanical ventilation duration and administration of aminoglycosides, glycopeptides, furosemide and dexamethasone were recorded. Aminoglycosides, glycopeptides and furosemide therapy was detailed as follows: number of cycles lasting at least three days and overall duration for each drug. The length of NICU stay was also registered.

PMA is the elapsed time between the first day of the last menstrual period and birth (GA) plus the elapsed time after birth (chronological age).

CA is the word most appropriately used to describe children who were born preterm up to 2 years of age. CA is calculated by subtracting the number of weeks born before 40 weeks of gestation from the chronological age.

All data were retrospectively collected from medical records by a team of neonatologists. Data were then entered into an electronic data collection system (Research Electronic Data Capture [REDCap]). Participant confidentiality was maintained by using a numerical code assigned by the study coordinator.

### 2.5. Hearing Screening and Diagnostic Methods

**Figure 1:**
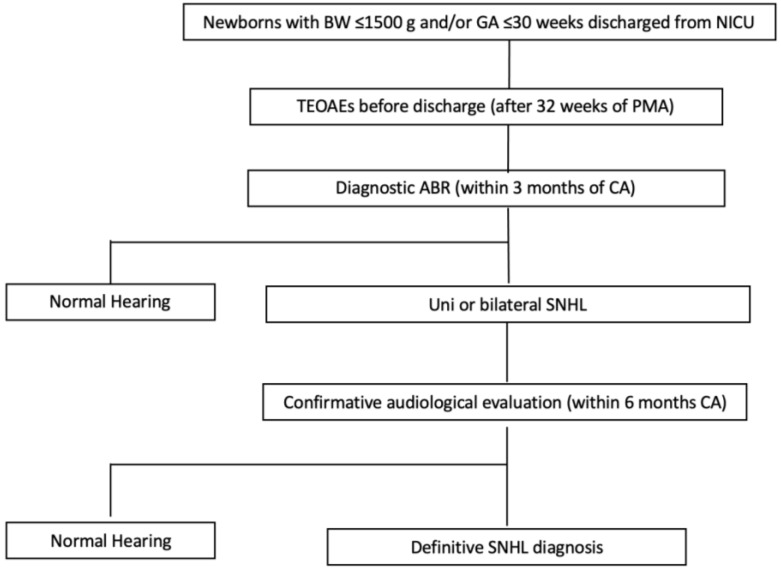
Hearing screening program applied in our study.

A schematic overview of the neonatal hearing screening program for our NICU infants is shown in Figure 1. The audiological evaluation consisted of medical history, otoscopy, TEOAEs, diagnostic (laboratory) ABR recording and, if necessary, tympanometry.

TEOAEs recording was performed before discharge and after 32 weeks of PMA using the Madsen Accuscreen^®^ (Natus Medical Incorporated, Orlando, FL, USA) which performs an evaluation of a patient’s TEOAES through a noise-weighted averaging and counting of significant signal peaks. The stimulus consisted of a non-linear click sequence at a rate of 60 Hz, which was delivered through the probe at a sound pressure level of 70–84 dB SPL, with a self-calibration depending on ear canal volume.

The first ABR recording was performed within 3 months of CA in a soundproof and electrically shielded room. The condition of natural sleep or quiet throughout the recording session was monitored in each infant by visual inspection and EEG signal. Both ears were sequentially tested. Stimuli were 0.1 ms clicks presented with alternating polarity by earphones (TDH-49P) at a rate of 21.1/s. The recording system was an ICS Chartr EP equipped with an ICS Chartr PA-800 preamplifier. Surface electrodes were placed at the vertex (+), ipsilateral ear lobe (−) and contralateral ear lobe (ground) and inter-electrode impedance was kept under 5 kΩ. The signal was amplified (100 k) and filtered (50–3000 Hz). Each trace was obtained by averaging 500–1500 single epochs and was replicated at least twice, mainly at the electrophysiological threshold level. The latter was determined as the lowest intensity level where a response could be assessed by the identification and replicability of the V wave. Starting from 60 dBnHL level, the threshold was assessed by a “20 dB down–10/5 dB up” procedure. At the end of the session, a recording at 80 dBnHL was usually performed for each side, allowing a better evaluation of morphology and latency of the ABR response. ABR recording and analysis were performed by an audiology technician under the supervision of an expert physician. When required, a full audiological assessment tympanometry and stapedius reflex thresholds were achieved by means of a Tympstar Grason-Stadler^®^ (Eden Prairie, MN, USA) with a 660 Hz probe tone delivered at 85 dB SPL ± 1.5 dB in all cases.

Infants affected by unilateral or bilateral hearing loss (UHL, BHL) were addressed with follow-up and further audiological evaluations until a definitive diagnosis was achieved within 4–6 months of CA. Possible changes in hearing threshold were also carefully evaluated for at least 12 months of CA with periodic audiological assessments.

The hearing threshold (dB HL) was estimated by subtracting an amount of 10 dB from the electrophysiological threshold (dBnHL) [19]. Hearing loss was then defined as mild (20–40 dB), moderate (41–70 dB), severe (71–90 dB) or profound (>90 dB) [20].

### 2.6. Sample Size and Statistical Analysis

The total number of births in Italy is about 400,000 per year, while the incidence of preterm births (GA < 37 weeks) is reported to be about 7%, so the expected premature infants are about 28,000 per year [21].

Since the incidence of SNHL among preterm infants is reported to be approximately 4% [1], a sample size of 59 preterm infants per year was needed to find the incidence of SNHL, with a 5% margin of error and a 95% confidence interval [22]. Since the study was planned for 5 years, at least 295 VLBW infants were expected to be included in the study.

With regard to statistical analysis, continuous variables were presented as mean ± SD and categorical variables as numbers and percentages. Comparisons between continuous variables were performed using Student’s *t*-test or Wilcoxon rank-sum test as appropriate. Comparisons between categorical variables were performed using Fisher’s exact test. Logistic regression analysis was used to further analyze loop diuretic and antibiotic therapy with regards to SNHL risk; and crude OR and OR corrected by GA with CI95% were reported.

A two-tailed *p* < 0.05 was considered as significant. Statistical analyses were performed with Stata 14 for Windows.

## 3. Results

From January 2012 to December 2016, 324 infants with BW < 1500 g were admitted to our NICU, and they were all screened through TEOAEs after 32 weeks of PMA and before discharge and through ABR within 3 months of CA. The parents of eight infants refused to participate in the study; therefore, 316 infants were enrolled. SNHL was diagnosed in 68 infants, leading to an incidence of 21.5%, while 248 infants were found to be without SNHL.

Infants with SNHL had significantly lower GA and BW and were sicker than infants without SNHL. Infants who experienced hearing loss also had a more complicated NICU stay: they had a higher incidence of sepsis, brain injury, BPD and surgical ROP than normal hearing babies; moreover, they underwent more gastrointestinal surgery and were hospitalized longer (Table 1).

Looking at the potential ototoxic therapies most frequently used in our NICU, infants with SNHL had significantly more days of mechanical ventilation and oxygen therapy and received more ototoxic drugs, such as aminoglycosides, glycopeptides and furosemide (Table 2).

Based on the logistic regression analysis, only the number of cycles of aminoglycosides and glycopeptides and the overall duration of these antibiotic therapies significantly increase the odds of SNHL, even after correction of OR by GA (Table 3).

**Figure 2:**
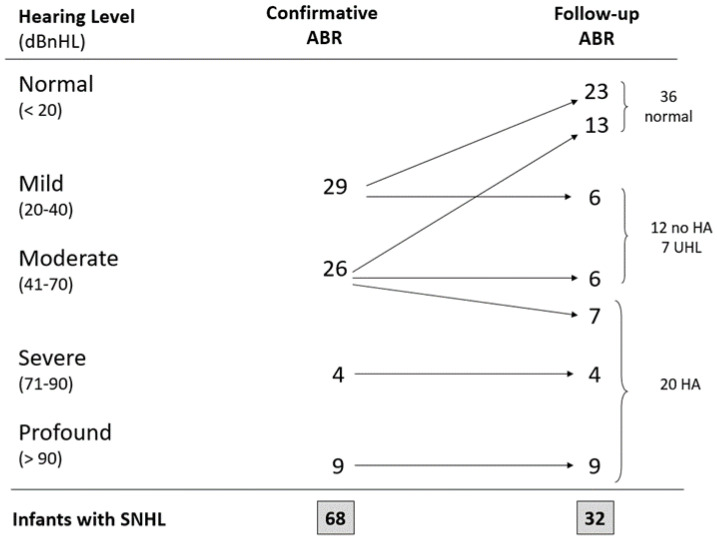
Audiological features and follow-up in infants with SNHL.

Figure 2 shows audiological findings at the time of diagnosis and at follow-up in the 68 infants with SNHL. At the first ABR evaluation within 3 months of CA, 29 of them had mild, 26 had moderate, 4 had severe, and 9 had profound hearing loss. At the final evaluation within 12 months of CA, 36 out of 68 (53%) with the initial finding of SNHL recovered normal hearing.

In 12 babies, 6 with mild SNHL (5 bilateral and 1 unilateral) and 6 with moderate UHL, the following checks confirmed the early diagnosis and these babies were enrolled into audiological and phoniatric monitoring. Depending on monitoring results and in accordance with parental opinion, in 3 out of 6 cases with moderate UHL, speech therapy was then activated.

The previous diagnosis of bilateral SNHL was confirmed in 20 infants (20/316, 6.3%), in which hearing threshold (7 moderate, 4 severe, 9 profound) did not change during follow-up. Definitive diagnosis was reached by the fifth month of CA and hearing aids (HA) were fitted at the age of 5.5 ± 1.3 months of CA. In 3 of these patients, a cochlear implant was later indicated. No case of auditory neuropathy spectrum disorder was found.

Infants who needed HA were significantly smaller and sicker and had a significantly longer stay in NICU in comparison with infants who did not require amplification (Table 4).

Moreover, infants needing HA received a higher therapy of cycles of furosemide, aminoglycosides and glycopeptides, as well as a longer duration of oxygen therapy and invasive mechanical ventilation (Table 5).

At last, we enrolled 316 infants and SNHL was diagnosed in 68. Out of these 68 infants, 36 infants improved. Finally, SNHL was confirmed in 20 patients (6.3%) who needed hearing aids. These infants were significantly smaller, sicker, had longer hospitalization and received more ototoxic therapies. Logistic regression analysis showed how gestational age (GA) influenced the association between drugs and SNHL. The total exposure to antibiotics is significantly associated with SNHL, even after GA correction.

## 4. Discussion

Our study aimed to investigate the prevalence and the severity of hearing loss and the related risk factors in a population of VLBW infants.

Since we considered every degree of HL as well as unilateral forms, the overall prevalence of HL was higher than in other reports [8,13]. The hearing-impaired infants, which were addressed to amplification, were 20 out of 316 (6.3%), and this finding is in agreement with some previous research [1,3,11].

No infants were lost to follow-up and this strengthened the quality of our hearing screening program and allowed us to perform a reliable analysis of data.

Assessment of risk factors for SNHL is challenging and JCIH guidelines addressed to the general population could not be completely appropriate for high-risk preterm infants.

We considered JCIH criteria as well as additional risk factors based on up-to-date literature to evaluate treatment modalities in the NICU setting as effective risk factors.

The prevalence of permanent SNHL in newborns admitted to NICU was almost seven times higher in comparison with normal babies [1]. Prolonged assisted ventilation and oxygen therapy, ototoxic medications, asphyxia, hyperbilirubinemia and high noise levels are associated with hearing loss related to NICU stay [3,8,23]. In addition, VLBW infants have higher and additional risk [1]; for instance, Wroblewska-Seniuk et al. showed that the prevalence of hearing loss is inversely related to GA [3]. We observed that hearing impaired infants have been hospitalized for longer time in our NICU and showed a greater morbidity than infants without SNHL.

Hypoxia was strongly associated with SNHL since it causes irreversible cellular damage to the cochlea, even though there is no clear threshold level at which auditory development is at risk. Low Apgar score at 1 and 5 min was strongly associated with SNHL [3,12] but, in our population, we did not find a significant difference between the two groups with regard to Apgar score. However, our infants with hearing impairment required more days of mechanical ventilation and oxygen therapy and both treatments could be considered as a proxy for hypoxic damage. Furthermore, it should be emphasized that mechanical ventilation and oxygen therapy hold more risk factors associated with SNHL like any prescription of ototoxic therapy, the presence of related pathologies, such as infections or BPD, and main surgery.

The association between sepsis and HL is also still debated [3,23] because sepsis includes multiple risk conditions for SNHL. However, we observed that, in our population, sepsis is significantly more frequent in infants with hearing impairment.

Ototoxic drugs, such as antibiotics and loop diuretics, have been significantly associated with the development of SNHL [8,24]. Aminoglycosides are often used as the first line antibiotic treatment in newborns and are widely used in NICU, and it is well known that these agents can damage both vestibular and cochlear organs, producing temporary or permanent hearing loss [3,25]. The administration of aminoglycoside therapy is particularly dangerous for preterm infants in a noisy NICU [2]. Furthermore, loop diuretics can damage the cochlea and produce hearing loss and can also potentiate an aminoglycoside toxic effect [25]. However, this aspect is still debated. In our hearing impaired patients, the overall duration (days) of all ototoxic drugs as well as the number of times these drugs were prescribed (number of cycles) were significantly higher than in normal hearing patients. These findings are not in agreement with those of Wroblewska-Seniuk [3], who did not find a significant correlation between exposure to ototoxic medications and HL in a nation-wide premature newborns study. Similarly, Salvago et al. [26] did not find a relation between HL and ototoxic drugs in a study involving a heterogeneous population of preterm and at-term infants in which all ototoxic drugs are considered together and without specifying the overall duration of therapy.

Furthermore, Chant et al. [24], in agreement with our data, found that children with HL had lower BW and more severe neonatal illness. The duration of gentamicin, vancomycin and furosemide administration in the first 14 days was associated with impaired hearing, but their sequential multiple regressions showed how prematurity adds a further audiological risk. These authors did not report the age of diagnosis of HL, the audiological follow-up and the hearing threshold trends.

The latest JCIH guidelines [7] confirmed aminoglycoside administration as a risk factor for HL but only for treatment longer than 5 days, apart from toxic blood levels and a known genetic susceptibility. Other ototoxic drugs, despite being usually used in NICUs, were not considered at all.

The results of our logistic regression analysis highlight the role of GA in the association between drugs and SNHL. The inclusion of GA removed the effect of furosemide on hearing loss. On the contrary, the total entity of antibiotics (days and cycles) is significantly associated with SNHL even after GA correction. It is known that aminoglycoside toxicity appears to be correlated with pharmacokinetics, duration of treatment, concurrent ototoxic drugs, disease states and previous exposure to aminoglycoside [25]. Our data show how the immature cochlea appears to be more susceptible to repeated cycles of antibiotics (not only of aminoglycosides but also of glycopeptides). Unfortunately, we did not have data about serum peak and through concentrations, even if we always used therapeutic dosages according to the guidelines. Moreover, one should keep in mind that the availability of correct plasma levels of antibiotics does not rule out the possible combined action of other ototoxic events [25,27].

Repeated cycles of aminoglycoside therapy are significantly associated with SNHL and our population also includes infants who received aminoglycosides during rule-out sepsis protocols (three-day short therapy). The administration of aminoglycosides for less than 5 days [7] could give a false sense of security. It is always necessary to evaluate the real need for therapy to verify the simultaneous administration of other ototoxic drugs and the correct knowledge of the underlying pathology, especially in very preterm infants.

Our study highlights how the total entity of aminoglycoside exposure is significantly correlated with SNHL, while no association was found with the administration of the drug itself. It is known that polymorphisms in the mitochondrial gene MTRNR1 lead to an idiosyncratic HL after aminoglycoside administration unlike the ototoxicity related to sustained therapy. Although in our series no screening for mitochondrial DNA mutations was conducted, it should be emphasized that MTRNR1 mutations are an uncommon cause of SNHL in the Italian population, where there is a low prevalence of these polymorphisms [28]. Moreover, an analysis of genetic etiological factors for HL should be taken into consideration, although we cannot ignore the economic and organizational aspects. Moreover, we should consider that the prognostic value of this evaluation is more relevant from an audiological point of view rather than a neonatological one.

Neonatal HL is the second developmental disability [29] and the incidence of SNHL remains high, especially in preterm infants, despite the improvements in neonatal care. Nevertheless, there are no uniform and specific guidelines regarding hearing screening programs in premature babies and for timing of the conclusive audiological diagnosis [13].

JCIH 2007 recommended that all infants should have a hearing screening within 1 month of birth, comprehensive audiological evaluation no later than 3 months of age and HA fitting within 6 months of birth, when indicated. Complying with these indications in extremely preterm infants could imply that audiological diagnosis is performed too early and could lead to an overestimation of hearing dysfunction [30,31,32].

As already stated in our previous paper [33] and in contrast with proposals of other authors [3,23,26], we believe that 3 months of CA is an adequate age to achieve an ABR diagnosis, above all in extremely preterm infants. This behavior could allow to differentiate among changes in threshold due to the normal maturation of the auditory pathways, temporary HL and permanent SNHL needing treatment.

Moreover, we carefully analyzed changes in hearing threshold in the course of follow-up. Thirty-six infants out of 68 (53%), with the initial findings of SNHL, presented an improvement in their hearing threshold and recovered normal hearing. Apart from differences in study design and population, this behavior was already highlighted in several previous papers [30,34].

Confirmed and stable HL were found in our infants with significantly lower GA (always ≤27 weeks) and BW and with a longer and more complex NICU stay. In these infants, SNHL acoustic amplification by means of HA was activated within 6 months of CA, as strongly and widely proposed [20].

Unilateral hearing losses were also included in our study. UHL was found in 28 out of 316 infants (8.86%) at 3 months of CA, and this rate decreased to 2.2% (7/316) in later controls. Speech therapy was then activated in three infants with permanent UHL, thus confirming the need to address these patients with audiological follow-up [35].

JCIH in 2019 reviewed the risk factors for SNHL and does not specifically emphasize the role of BW less than 1250 g and premature delivery.

Audiological risk in these infants should be regarded as a problem not only of “immaturity”, but also as the result of multiple risk factors for hearing impairment, which can act in a combined and synergistic, synchronous and/or asynchronous way.

To reduce audiological risk for SNHL, we should operate on several aspects at the same time:

1. Reduce hospitalization in NICU and move infants as soon as possible to hospital departments with lower audiological risk.
2. Minimizing the use of antibiotics, loop diuretics and other ototoxic drugs, especially when used in combination.
3. Shortening the response times of cultures and implementation of microbiological surveillance programs in order to make the charge of antibiotic therapy more appropriate (36–48 h short therapy).
4. Control and try to reduce the ambient sound in the NICU.
5. 24 h presence of parents in the NICU because exposure to the maternal sounds and voice would have a role in reducing the frequency of apnea and bradycardia episodes, with a favorable impact on the development of the auditory system (and, therefore, of language) [36].

Limitations of our study are the retrospective and monocentric nature and the lack of pharmacokinetic data on ototoxic drugs. But, it also has several strengths: a sample size powered to find SNHL in VLBW infants and their risk factors; a homogeneous population of VLBW infants with similar neonatal care and treatments; data collected from medical records, not through interviews or questionnaires; and a complete audiological follow-up with no infants lost.

## 5. Conclusions

The specific knowledge of incidence of HL and risk factors allows the check of the quality of NICU care.

The results of or study underlined how GA, birth weight and, above all, the length and complexity of NICU stay quantify the risk of HL and should be considered in every single case for parent counseling. The total exposure to antibiotics is significantly associated with HL, even after GA correction.

Furthermore, we should take into account that a better knowledge of specific iatrogenic (therapeutic and/or environmental) risk factors could allow the implementation of the targeted bundle for the primary prevention of HL.

## Figures and Tables

**Table 1:** Baseline and clinical features of infants with and without SNHL.

	Infants with SNHL *n* 68	Infants without SNHL *n* 248	*p*
**GA, weeks**	27.6 ± 2.4	29.1 ± 1.5	<0.001
**BW, grams**	961.1 ± 350.4	1155.0 ± 283.9	<0.001
**Male gender**	40 (58.8)	115 (46.4)	0.075
**SGA**	11 (16.2)	26 (10.5)	0.2
**Z-score**	0.0 ± 1.1	0.1 ± 1.0	0.7
**1-min Apgar score**	5.8 ± 2.1	6.3 ± 1.8	0.1
**5-min Apgar score**	7.8 ± 1.4	8.1 ± 1.2	0.1
**Sepsis**	24 (35.3)	45 (18.1)	0.0044
**IVH > 2 grade/PVL** **≥** **2 grade**	18 (26.5)	36 (14.5)	0.028
**Major gastrointestinal surgery**	26 (36.7)	18 (7.2)	<0.001
**hsPDA**	3 (4.4)	3 (1.2)	0.11
**Surgical ROP**	13 (19.1)	3 (1.2)	<0.001
**Peak total bilirubin**	9.6 ± 2.7	9.4 ± 2.3	0.6
**BPD**	16 (23.5)	17 (6.8)	<0.001
**Length of NICU stay, days**	95.9 ± 59.5	56.1 ± 29.0	<0.001

**Table 2:** Potential ototoxic therapies in infants with and without hearing loss.

	Infants with SNHL *n* 68	Infants without SNHL *n* 248	*p*
**Aminoglycosides therapy, days**	23.2 ± 18.5	11.7 ± 9.9	<0.001
**Aminoglycosides therapy, n of cycles**	3.1 ± 1.7	2 ± 1.3	<0.001
**Furosemide therapy, days**	3.0 ± 6.9	1.9 ± 7.9	<0.001
**Furosemide therapy, n of cycles**	0.5 ± 1	0.2 ± 0.6	<0.001
**Glycopeptides therapy, days**	24.3 ± 25.4	9.8 ± 12.6	<0.001
**Glycopeptides therapy, n of cycles**	2.7 ± 1.9	1.5 ± 1.5	<0.001
**Exchange transfusion, n**	2 (2.9)	3 (1.2)	0.6
**Invasive mechanical ventilation, days**	15.0 ± 23	2.8 ± 7.8	<0.001
**Oxygen therapy, days**	32.2 ± 52	9.6 ± 23.1	<0.001

Data are reported as mean ± SD or number (percentage).

**Table 3:** Logistic regression analysis for ototoxic drugs on SNHL risk.

	Crude OR	OR Corrected by GA
**Furosemide**		
**Per prescription**	3.61 [1.91–6.84]	1.69 [0.81–3.51]
**Per number of cycles**	1.62 [1.18–2.24]	1.23 [0.87–1.73]
**Per number of days**	1.02 [0.99–1.05]	0.99 [0.96–1.03]
**Aminoglycosides**		
**Per prescription**	2.04 [0.69–6.02]	0.92 [0.29–2.88]
**Per number of cycles**	1.68 [1.38–2.03]	1.33 [1.06–1.68]
**Per number of days**	1.06 [1.04–1.09]	1.04 [1.02–1.07]
**Glycopeptides**		
**Per prescription**	2.7 [1.35–5.42]	1.43 [0.67–3.06]
**Per number of cycles**	1.52 [1.29–1.79]	1.23 [1.01–1.5]
**Per number of days**	1.04 [1.03–1.06]	1.02 [1.01–1.04]

**Table 4:** Baseline and clinical features of infants with and without HA.

	Infants with HA *n* 20	Infants without HA *n* 48	*p*
**GA, weeks**	25.6 ± 1.6	28.4 ± 2.2	<0.001
**BW, grams**	659.5 ± 152.5	1081.4 ± 334.3	<0.001
**Male gender**	10 (50.0)	29 (60.4)	0.42
**SGA**	5 (25.0)	6 (12.5)	0.2
**Z-score**	−0.4 ± 1.1	0.1 ± 1.1	0.07
**1-min Apgar score**	5.5 ± 1.8	6.1 ± 2.1	0.1
**5-min Apgar score**	7.8 ± 1.3	7.9 ± 1.1	0.9
**Sepsis**	11 (55.0)	13 (27.1)	0.049
**IVH >2 grade/PVL ≥ 2 grade**	10 (50.0)	8 (16.7)	0.0072
**Major gastrointestinal surgery**	11 (55.0)	15 (31.2)	0.09
**hsPDA**	3 (15.0)	0	0.023
**Surgical ROP**	10 (50.0)	3 (6.3)	<0.001
**Peak total bilirubin**	9.8 ± 2.6	9.5 ± 2.3	0.3
**BPD**	7 (35.0)	9 (18.8)	0.2
**Length of NICU stay, days**	154.3 ± 58.4	71.1 ± 40.4	<0.001

Data are reported as mean ± SD or number (percentage).

**Table 5:** Potential ototoxic therapies between infants with and without indication of HA.

	Infants with HA *n* 20	Infants without HA *n* 48	*p*
**Aminoglycosides therapy, days**	42.9 ± 18.3	15 ± 10.9	<0.001
**Aminoglycosides therapy, n of cycles**	4.8 ± 0.6	2.5 ± 1.5	<0.001
**Furosemide therapy, days**	6.2 ± 9.7	1.8 ± 5	<0.001
**Furosemide therapy, n of cycles**	1.3 ± 1.5	0.2 ± 0.5	<0.001
**Glycopeptides therapy, days**	52.7 ± 25.9	12.5 ± 12.8	<0.001
**Glycopeptides therapy, n of cycles**	4.7 ± 0.7	1.8 ± 1.5	<0.001
**Exchange transfusion, n**	1 (5.0)	1 (2.0)	0.5
**Invasive mechanical ventilation, days**	38.4 ± 27.9	5.2 ± 10.7	<0.001
**Oxygen therapy, days**	80.5 ± 70.9	12.1 ± 20.7	<0.001

## Data Availability

Datasets generated and/or analyzed during the current study are available from the corresponding author upon reasonable request.
